# Lycorine attenuated proliferation and induced apoptosis on imatinib-resistant K562 cell by inhibiting autophagy

**DOI:** 10.1007/s12672-024-01080-3

**Published:** 2024-06-10

**Authors:** Jun Bai, Zuxi Feng, Yaqiong Chen, Yanhong Li, Liansheng Zhang, Lijuan Li

**Affiliations:** 1 Department of Hematology, Lanzhou University Second Hospital, Lanzhou, 730000 China; 2 Gansu Province Hematologic Disease Clinical Medical Research Center, Lanzhou, 730000 China; 3 School of Basic Medical Sciences, Lanzhou University, Lanzhou, 730000 China

**Keywords:** Lycorine, Chronic myeloid leukemia, Imatinib drug resistance, Autophagy, Apoptosis, Cycle arrest

## Abstract

**Background:**

Tyrosine kinase inhibitor (TKI) resistance is a significant factor exacerbating the burden on chronic myeloid leukemia (CML) patients and impacting clinical efficacy. The main goal is to offer new insights into overcoming drug resistance in treating CML.

**Methods:**

Imatinib (IM) resistant K562/IM cells were generated using gradient induction. Responses to IM, lycorine, and autophagy modulators were assessed using CCK-8. Protein expression of Beclin-1, Atg5, LC3, Caspase-3, P62, Bax, Bcl-2, and P-gp was detected using Western blot. Lycorine-induced apoptosis and cell cycle changes were evaluated through flow cytometry, while autophagy alterations were detected using monodansylcadaverine (MDC) staining. In the K562/IM mice model, non-obese diabetic severe combined immunodeficent (NOD-SCID) mice were subcutaneously inoculated with K562/IM cells. After 17 days of lycorine injection, assessments included tumor size, hematoxylin–eosin (HE) staining, and Ki67 expression.

**Results:**

After 72 h of IM treatment, K562/IM cells showed a 55.86-fold increase in drug resistance compared to K562 cells. Lycorine treatment for 24 h inhibited cell proliferation and induced G0/G1 phase cell cycle arrest and apoptosis in both K562 and K562/IM cells. MDC staining indicated reduced autophagy in K562/IM cells, mitigated by lycorine. In vivo experiments demonstrated reduced tumor size and Ki67 proliferation index in the lycorine treatment group (K562+L, K562/IM+L) compared to the control group, particularly in the drug-resistant group. However, no significant change in Ki67 was observed in the K562 group after lycorine treatment.

**Conclusion:**

In summary, K562/IM cells displayed heightened autophagy levels compared to K562 cells. Lycorine effectively impeded the proliferation of K562/IM cells through diverse mechanisms, including reduced autophagy, enhanced apoptosis, and induced cell cycle arrest.

**Supplementary Information:**

The online version contains supplementary material available at 10.1007/s12672-024-01080-3.

## Introduction

CML is a hematologic malignancy characterized by the clonal proliferation of hematopoietic stem cells, constituting 15-20% of all adult leukemia cases [1]. CML progresses through distinct phases—chronic, accelerated, and acute. Annually, approximately 3% to 4% of individuals in the chronic phase transition to the acute phase, with a median duration of 6 to 10 months [2]. The primary clinical intervention for CML involves the administration of a TKI, proving efficacious in enhancing survival rates by mitigating the acceleration and abrupt alterations characteristic of CML progression [3]. Resistance to TKIs is a significant concern, necessitating thorough examination, as the precise mechanisms remain elusive and require comprehensive elucidation.

A growing body of evidence establishes a close association between autophagy and the manifestation of various diseases, including cancer, neurodegenerative disorders, diabetes, autoimmune conditions, and cardiovascular ailments [4]. Research findings indicate a correlation between autophagy and TKI resistance, where upregulation of the Beclin-1 and ATG-5 genes renders TKIs unable to induce apoptosis in CML cells. Autophagy, acting as a protective mechanism, leads to the development of drug resistance [5]. Consequently, autophagy serves as a protective mechanism for certain CML cells, ultimately resulting in the development of drug resistance. Scientists employ autophagy inhibitors, compounds, and genetic engineering techniques to attenuate the expression of autophagy-related genes, aiming to enhance the apoptotic demise of CML cells. Clinical investigations, targeting solid tumors and hematopoietic malignancies, including CML, chronic lymphocytic leukemia, and multiple myeloma, have been conducted, involving the co-administration of autophagy inhibitors with chemotherapeutic agents [6]. However, existing autophagy inhibitors, like hydroxychloroquine, face limitations in achieving optimal plasma concentrations, necessitating the pursuit of enhanced autophagy inhibitors to address TKI resistance at its foundational level [7]. Yet, the prognosis for patients in the acute phase of CML following treatment with autophagy inhibitors is discouraging [8].

Lycorine, a monomeric compound derived from traditional Chinese medicine, is an active isoquinoline alkaloid found in Amaryllidaceae plants [9]. In a study by L Li et al., lycorine was identified as reducing the survival rate of leukemia HL-60 cells and myeloma KM3 cells, inducing apoptosis [10]. Lycorine's autophagy-inhibitory properties were observed, as it reduced lipopolysaccharide-induced autophagy through a decrease in LC3-II and an increase in P62. This reduction in autophagy subsequently decreased both the number and activity of osteoclasts, mitigating associated symptoms [11]. Therefore, we propose exploring the application of lycorine in treating CML-resistant patients, aiming to assess its efficacy in autophagy inhibition and delve into the underlying regulatory mechanisms. Such an investigation could potentially serve as a breakthrough in alleviating or reversing TKI resistance.

## Materials and methods

### Cell Lines and reagents

The K562 human myeloid leukemia cell line was procured from Guangzhou Saiku Biotechnology Co., Ltd. Cultivation of the cells took place in 1640 medium (US, Gibco, C11875500BT), supplemented with Chinese-origin fetal bovine serum (Uruguay, Abwbio, AB-FBS-1050S). Phosphate-buffered saline (PBS) (China, BasalMedia, B320KJ) was employed for buffer preparation. Key reagents, including Trizol pyrolysis solution, Green/streptomycin, and CCK-8, were sourced from Solarbio (China, R1100), AbMole (US, p1400), and Solarbio (China, m4839), respectively. Antibody dilution was performed using New Saimei (China, WB100D) products, and Glycine was obtained from Solarbio (China, G8200). The PMSF protease inhibitor was acquired from Biyuntian (China, ST505). Various antibodies were utilized: Goat anti-rabbit secondary antibody (SA00001-2), Beclin-1 rabbit polyclonal antibody (26,593-1-AP), Beta-actin rabbit polyclonal antibody (20,536-1-AP), Atg5 rabbit polyclonal antibody (10,181-2-AP), Caspase3 rabbit polyclonal antibody (19,677-1-AP), and LC3 rabbit polyclonal antibody (14,600-1-AP), all from Proteintech, US. RIPA cell lysate was sourced from Solarbio (China, r0010), and Monodansylcadaverine was obtained from Sigma (US, 30,432). The enhanced chemiluminescence (ECL) hypersensitivity luminescence kit was procured from Yeasen (China, 36208ES60). Furthermore, cell cycle and apoptosis kits were acquired from Biyuntian (China, c1052). P-gp rabbit polyclonal antibody, BAX rabbit polyclonal antibody, P62 rabbit polyclonal antibody, and Bcl-2 rabbit polyclonal antibody were obtained from Proteintech (US, 22,336-1-AP, 50,599-2-lg, 18,420-1-AP, 26,593-1-AP). Imatinib and lycorine hydrochloride were obtained from Abmole (US, M3241, M4691). Hydroxychloroquine (HCQ), 3-Methyladenine (3-MA) and Rapamycin (RAPA) were obtained from Abmole (US, M5159, M2296, M1768). HE staining and Ki67 proliferation index staining were obtained from Solarbio (China, G1120) and Proteintech (US, 28,074-1-AP).

### Construction of IM-resistant cell line K562/IM

The K562 human myeloid leukemia cell line was cultured and sustained in a cell incubator set at 37 °C with a 5% CO_2_ atmosphere. The culture medium was replenished every 2–3 days, and passaging occurred upon reaching a cellular confluence of 70-80%. An IM solution was prepared by diluting IM in 1640 medium to achieve a final concentration of 100 μM, subsequently stored in a refrigerator at 4 °C for future use [12]. Employing a gradient induction approach, we established K562/IM with an initial IM intervention concentration of 0.1 μM. This concentration increased by 0.1 μM every 7 days, reaching an IM induction concentration of 1.0 μM after approximately 2 months. Subsequently, the intervention concentration escalated by 0.5 μM every 10 days. Over this period, the K562/IM cell line, stabilized at 3 μM IM resistance, was maintained and incrementally reached 20 μM. This drug was consistently incorporated into the culture of K562 cells for 10 months. Following the cessation of IM, the cells exhibited normal proliferation in a complete medium containing 5 μM IM. Standard passages were conducted, resulting in the development of K562/IM resistant to 5 μM IM.

### IM resistance index determination of K562/IM

The K562 and K562/IM cell lines were obtained from the cell culture incubator. Sterile 96-well plates were prepared, and cells were allocated to two groups: K562 and K562/IM. Subsequently, cells were dispensed into six parallel wells within each group after fluid exchange. After a 72-h incubation period at 37 °C in a 5% CO2 cell incubator, 20 µL of CCK-8 was added to each well. Following an additional 1-h incubation, the absorbance at 450 nm was measured using an enzyme marker. The determination of the half-lethal concentration (IC50) and the calculation of the resistance index (RI) were conducted [13]. The resistance index is defined as the IC50 of resistant cells divided by the IC50 of parental cells. An RI greater than 5 indicates the presence of drug resistance.

### CCK-8 assay for detection of cell proliferation activity

K562 cells and K562/IM cells were seeded in 96-well plates at a density of 5000 cells per well. The experimental groups comprised the following treatments: 5 μM IM, 1.5 μM lycorine, 3 μM lycorine, 6 μM lycorine, 6 μM lycorine with 100 nM RAPA, 6 μM lycorine with 5 mM 3-MA, 6 μM lycorine with 20 μM HCQ, and a Control group. Each group consisted of three parallel replicate wells. After a 24-h incubation period, 20 µL of CCK-8 was introduced into each well. Subsequently, following a 1-h incubation, the absorbance values at 450 nm were quantified using an enzymatic marker. To simplify, the group denoted as K562+L refers to K562 cells treated with 6 µM lycorine, while the group designated as K562/IM+L represents K562/IM cells treated with 6 µM.

### Sequencing and analysis

We utilized K562/IM and K562/IM+L cells for mRNA sequencing, involving three samples for each condition (K562/IM and K562/IM+L). In each case, 1×10^6 cells were dispersed in 1 mL of Trizol reagent. The mRNA sequencing, along with subsequent analysis, was performed by Shanghai Tianhao Biotechnology Co., Ltd.

### Detection of apoptosis and cycle changes

K562 and K562/IM cells in the logarithmic growth phase were harvested and exposed to various dosing conditions for a duration of 24 h. Subsequently, the harvested samples were meticulously prepared and stained in accordance with the procedural guidelines provided by the apoptosis kit, with apoptosis being evaluated through flow cytometry. In addition, Wright's staining was employed to analyze cell apoptosis rates 24 h after drug administration. For the specifically chosen K562 and K562/IM cells in the logarithmic growth phase, cell cycle distribution and blockade were examined using flow cytometry following a 24-h intervention under different dosing conditions. The samples were prepared and stained according to the operational instructions provided by the cell cycle kit.

### MDC fluorescence staining for detection of autophagy acidic vesicles

MDC powder was dissolved in dimethyl sulfoxide to generate a parent solution having a concentration of 50 mM [14]. Subsequently, this solution underwent further dilution with PBS to attain a concluding concentration of 0.05 mM, facilitating fluorescence staining with MDC. The experimental groups included a 6 μM lycorine treatment group and a control group. K562 cells and K562/IM cells in the logarithmic growth stage were chosen and cultured for 24 h under the specified drug interventions. Subsequently, the cells were stained with 1 mL of 0.05 mM MDC for 15 min and examined under an inverted fluorescence microscope to observe autophagic acidic vesicles. This procedure allowed for the identification and characterization of autophagic acid vesicles.

### Detection of protein expression of K562 after different drug intervention by western blot

K562 and K562/IM cells underwent a 24-h drug treatment to constitute the experimental group, with untreated cells serving as the control group. Following this intervention, total protein extraction was executed for each group, and the bicinchoninic acid (BCA) assay kit was employed for protein quantification. Subsequently, sample volumes were determined. The experimental procedure encompassed sodium dodecyl sulfate–polyacrylamide gel electrophoresis (SDS-PAGE) gel electrophoresis, polyvinylidene fluoride (PVDF) membrane transfer, skim milk blocking, primary antibody incubation, secondary antibody labeling, and fluorescence stimulation using the ECL luminescence solution. Following strip exposure, Image J software facilitated data processing, and GraphPad Prism software was utilized for graph analysis.

### In vivo experiment

In this study, we obtained twelve female mice belonging to the NOD-SCID immunodeficient mice, within the 3–5 weeks age range and a weight range of 18–20 g, was systematically assigned to one of four experimental groups: K562, K562/IM, K562-L, and K562/IM-L. The experimental protocols involved the utilization of K562 and K562/IM cell lines, with three mice allocated to each group. The mice were sourced from GemPharmatech Co., Ltd. located in Shanghai, China. A total of 1 × 10^7^ cells of either K562 or K562/IM in 150 μL PBS were subcutaneously administered into the right axilla of the mice. Animal experiments were approved by the Ethics Committee of the Lanzhou University Second Hospital. The mouse housing environment is maintained within a temperature range of 20 °C to 26 °C, with humidity levels between 30 and 70%, a 12-h light–dark cycle, and ample provision of drinking water and nutritionally balanced feed. Following inoculation, the dimensions of the resultant tumors were measured daily. Euthanasia criteria were established for mice exhibiting subcutaneous nodules exceeding a diameter of 18 mm or reaching 17 days post-tumor inoculation. Tumor volume was calculated using the formula (length × width^2^)/2. We confirm that the maximal tumor size/burden did not exceed the standards set by the Ethics Committee of Lanzhou University Second Hospital.

### Immunohistochemistry (IHC)

The xenograft tissues were meticulously processed, beginning with fixation in formalin, followed by paraffin embedding, deparaffinization, and hydration. Subsequently, HE staining was employed. Ki67 proliferation index staining analyses were conducted utilizing both primary and secondary antibodies, and the evaluation of IHC positivity employed the Fromowitz scoring method. Staining intensity was categorized as follows: 0 points denoted no staining, 1 point corresponded to pale yellow, 2 points represented brownish-yellow, and 3 points indicated brown staining. For each section, five randomly selected fields were scrutinized, and within each field, 100 cells were assessed. The proportion of positively stained cells was stratified as follows: < 5% was scored as 0, 5–25% as 1, 26–50% as 2, 51–75% as 3, and > 75% as 4. The final IHC score was derived by summing the scores from these two components.

### Statistical analysis

Statistical analyses were executed employing GraphPad Prism 8.0 software, with graphical representations also crafted using this software. Descriptive statistics are conveyed as mean values accompanied by their respective standard deviations. Group comparisons were executed through the application of the Student t-test and one-way ANOVA. To ascertain statistical significance, a significance threshold of P < 0.05 was applied. Levels of significance are denoted as follows: *P < 0.05; **P < 0.01; ***P < 0.001; ****P < 0.0001.

## Results

### Lycorine suppresses proliferation in K562 and IM-resistant K562/IM Cells

**Fig. 1:**
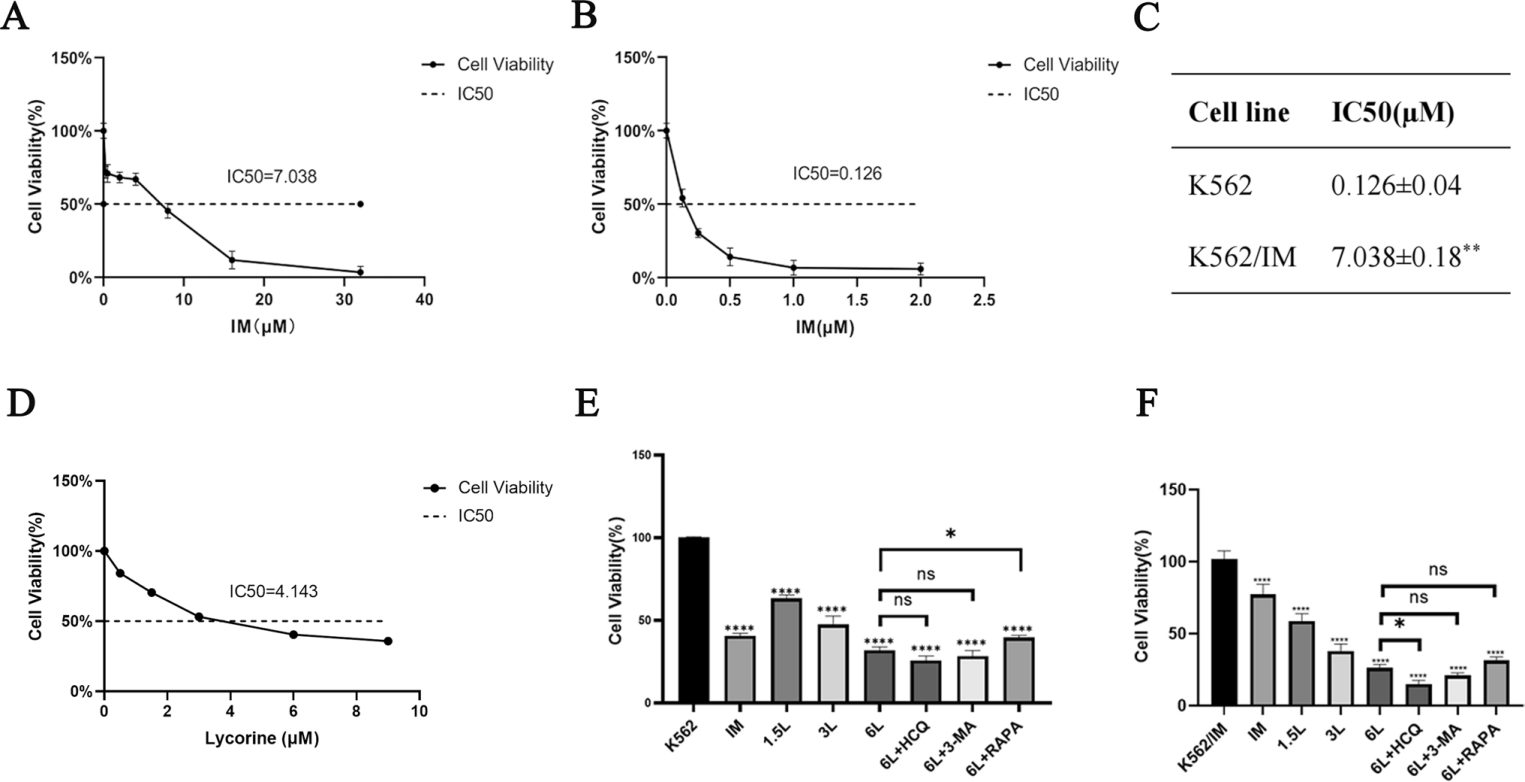
Lycorine inhibits K562 and K562/IM cell proliferation. The present study involved the assessment of cell viability in two distinct cellular strains: **A** The assessment of cell viability in the K562/IM resistant strain. Statistically significant differences were observed between these strains (P < 0.05). **B** The assessment of cell viability in the K562 normal strain. Statistically significant differences were observed between these strains (P < 0.05). To determine the half-inhibitory concentrations of K562 and K562/IM cells subjected to varying concentrations of IM over a 72-h period, the CCK-8 method was employed. **C** The IC50 of K562 cells served as a reference for comparison. **D** The cytotoxic effects of different concentrations of Lycorine on K562 cells. **E** The effects of different drug interventions on cell viability in K562 cells after 24 h. The drug groups include K562 group, IM group, 1.5 μM Lycorine (1.5L) group, 3 μM Lycorine (3L) group, 6 μM Lycorine (6L) group, 6 μM Lycorine (6L)+HCQ group, 6 μM Lycorine (6L)+3-MA group, and 6 μM Lycorine (6L)+RAPA group. **F** K562/IM cells treated with various drugs for 24 h were investigated. The drug groups include K562+IM group, IM group, 1.5 μM Lycorine (1.5L) group, 3 μM Lycorine (3L) group, 6 μM Lycorine (6L) group, 6 μM Lycorine (6L)+HCQ group, 6 μM Lycorine (6L)+3-MA group, and 6 μM Lycorine (6L)+RAPA group. *P < 0.05; **P < 0.01; ***P < 0.001; ****P < 0.0001

We assessed the drug sensitivity of wild-type K562 cells and IM-resistant K562/IM cells to varying concentrations of IM using the CCK-8 assay. Subsequently, we calculated the median lethal concentration (IC50) and drug resistance index. Significantly, the IM resistance of K562/IM cells exceeded that of K562 cells across varying IM concentrations after a 72-h exposure period (Fig. 1A–B). The resistance index of K562/IM cells was 55.86 times higher than that of K562 cells (Fig. 1C). Subsequently, we employed the CCK-8 assay to assess the cytotoxic impact of Lycorine on K562 cells. In comparison to the control group, the viability of K562 cells, subjected to diverse concentrations of lycorine, demonstrated a progressive, dose-dependent reduction over the course of the experiment (Fig. 1D). HCQ and 3-MA are autophagy inhibitors, suppressing the occurrence of autophagy. RAPA, on the other hand, is an autophagy activator, promoting autophagy by inhibiting the mTOR signaling pathway. Following treatment with 5 µM IM, 6 µM Lycorine, and a combination of HCQ/3-MA/RAPA for 24 h, a substantial reduction in cell viability was observed (Fig. 1E). In contrast to the control group, there was a notable reduction in cell proliferation across all experimental groups, exhibiting a more pronounced inhibitory effect as the concentration of Lycorine increased. In the K562 experiment, the cell survival of the Lycorine combined with RAPA group was higher than that of the Lycorine group. In the K562/IM experiment, the cell survival of the Lycorine combined with HCQ group was lower than that of the Lycorine group, with no statistical difference in other groups (Fig. 1F).

### Transcriptome sequencing analysis

**Fig. 2:**
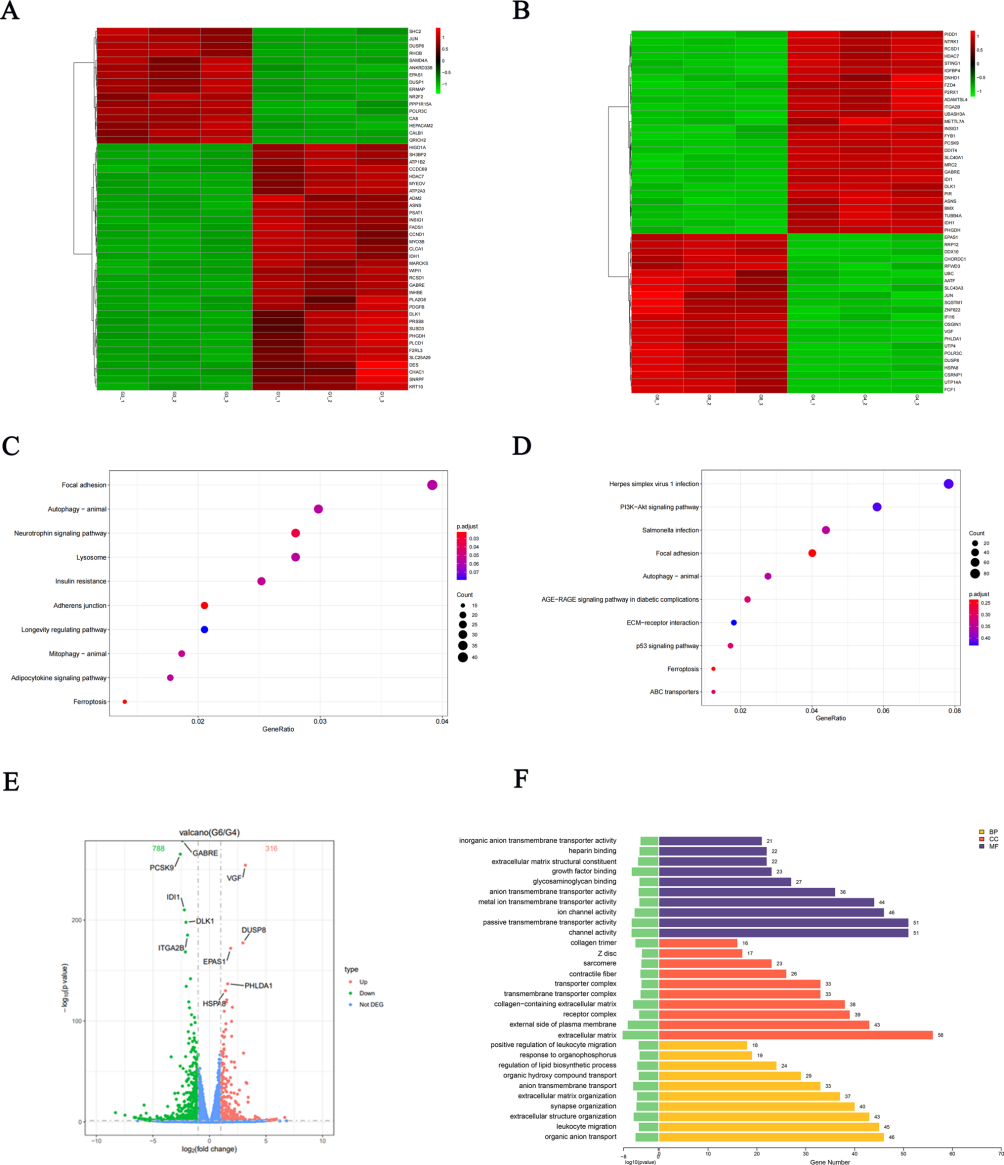
Transcriptome sequencing analysis. **A** a thermogram displaying clustering patterns of the top 50 differentially expressed mRNAs between K562 group and the K562+L group. **B** a thermogram displaying clustering patterns of the top 50 differentially expressed mRNAs between K562/IM group and the K562/IM+L group. **C** a bar graph presents the GO enrichment of differentially expressed genes between K562 group and the K562+L group. **D** a bar graph presents the GO enrichment of differentially expressed genes between K562/IM group and the K562/IM+L group. **E** a volcanic map depicting differential mRNA expression between K562/IM group and the K562/IM+L group. **F** a scatter plot illustrating the KEGG enrichment of differentially expressed genes between K562/IM group and the K562/IM+L group. Note: the experimental groups consisted of three samples,biological processes (BP), cell composition (CC), and molecular function (MF)

Transcriptome sequencing was employed to scrutinize the transcriptional profiles of K562, K562+L (6 µM), K562/IM and K562/IM+L (6 µM) cells. The clustering heatmap of differential mRNA (top 50) between K562 and K562+L group (Fig. 2A). The clustering heatmap of differential mRNA (top 50) accentuates a marked up-regulation of the autophagy-related gene SQSTM1 (P62) in the K562/IM+L group (Fig. 2B). Kyoto Encyclopedia of Genes and Genomes (KEGG) signal pathway enrichment analysis results indicate that the differentially expressed genes are predominantly associated with autophagy and autophagy-related PI3K/Akt pathway (Fig. 2C-D). A comparison between the K562/IM group and the K562/IM+L group revealed 104 genes exhibiting significantly altered expression in the latter, encompassing 316 up-regulated genes and 788 down-regulated genes (Fig. 2E). Moreover, the results of Gene Ontology (GO) biological function enrichment analysis delineate that the differentially expressed genes primarily cluster in biological processes such as passive transmembrane transporter activity, ion channel activity, outer plasma membrane, extracellular matrix, leukocyte migration, and organic anion transfer (Fig. 2F).

### Lycorine induced apoptosis of K562 and K562/IM cells

**Fig. 3:**
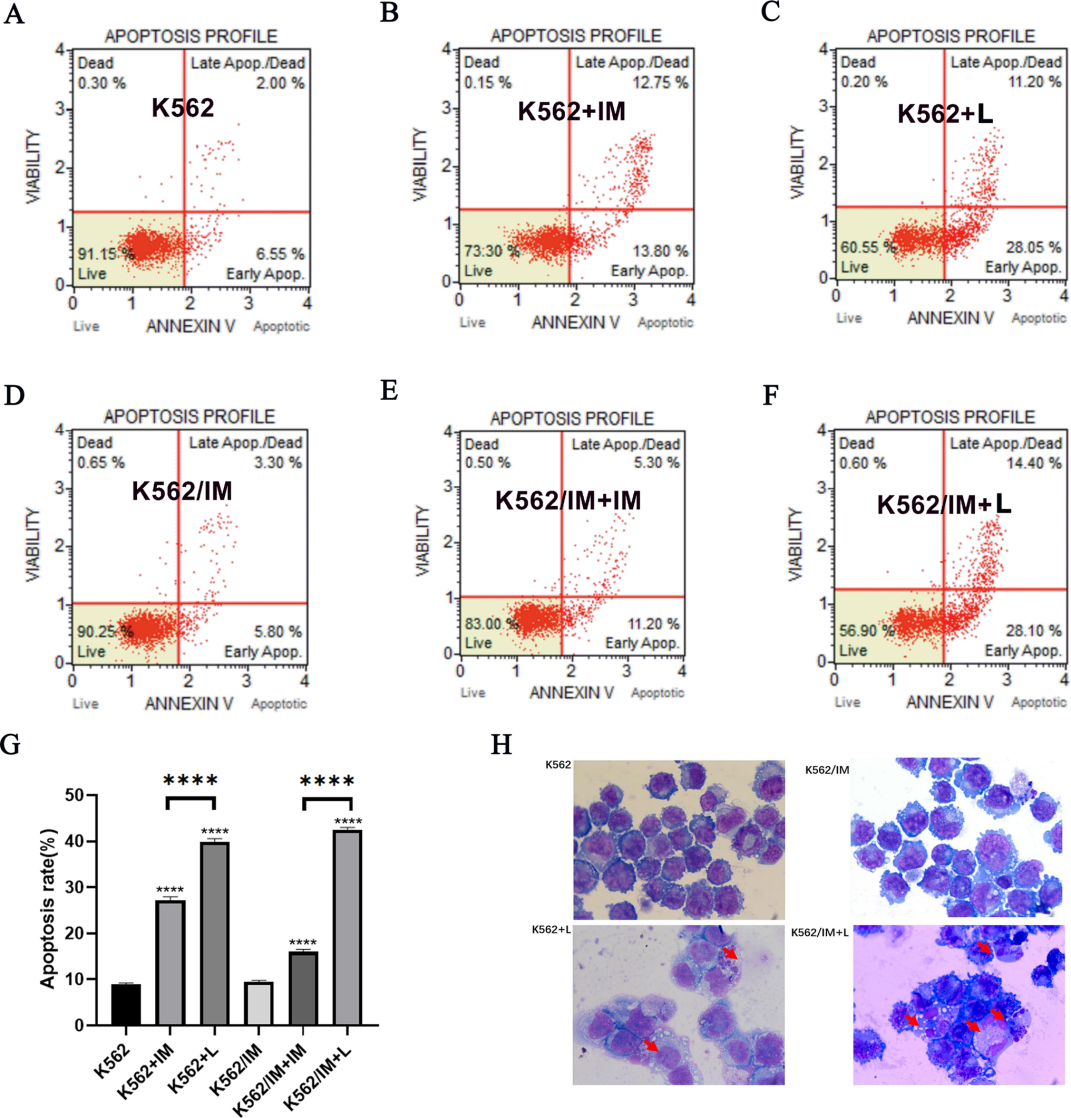
Lycorine induced apoptosis of K562 and K562/IM cells. **A**–**F** The flow cytometry results for apoptosis detection were displayed for the following treatment groups: K562 group, K562+IM group, K562+Lycorine (L) group, K562/IM group, K562/IM+IM Lycorine (6L) group, and K562/IM+Lycorine (L) group. **G** The statistical analysis of apoptosis rates across various dosage groups. **H** The observation of apoptotic bodies through Wright staining after a 24-h period. Notably, red arrows are employed to indicate apoptotic cells. *P < 0.05; **P < 0.01; ***P < 0.001; ****P < 0.0001

The evaluation of apoptosis in K562 and K562/IM cells after a 24-h treatment was carried out utilizing Annexin V/PI double staining flow cytometry. Following the application of lycorine (6 μM), a marked augmentation in apoptosis incidence was observed in both the K562 and K562/IM cohorts. Notably, K562 cells exhibited a significant surge in apoptosis following treatment with 5 μM IM, while K562/IM cells showed a modest increase post-IM treatment. The Lycorine group demonstrated a significantly higher apoptosis rate compared to each IM group, and this difference was statistically significant (Fig. 3A–G). For the assessment of the apoptosis ratio after a 24-h administration period, Wright's staining was employed, and the results are presented in Fig. 3H. In comparison to both the K562 group and the K562/IM group, there was a statistically significant increase in apoptotic bodies in K562 +L and K562/IM+L.

### Lycorine induced cell cycle arrest in both K562 and K562/IM cells

**Fig. 4:**
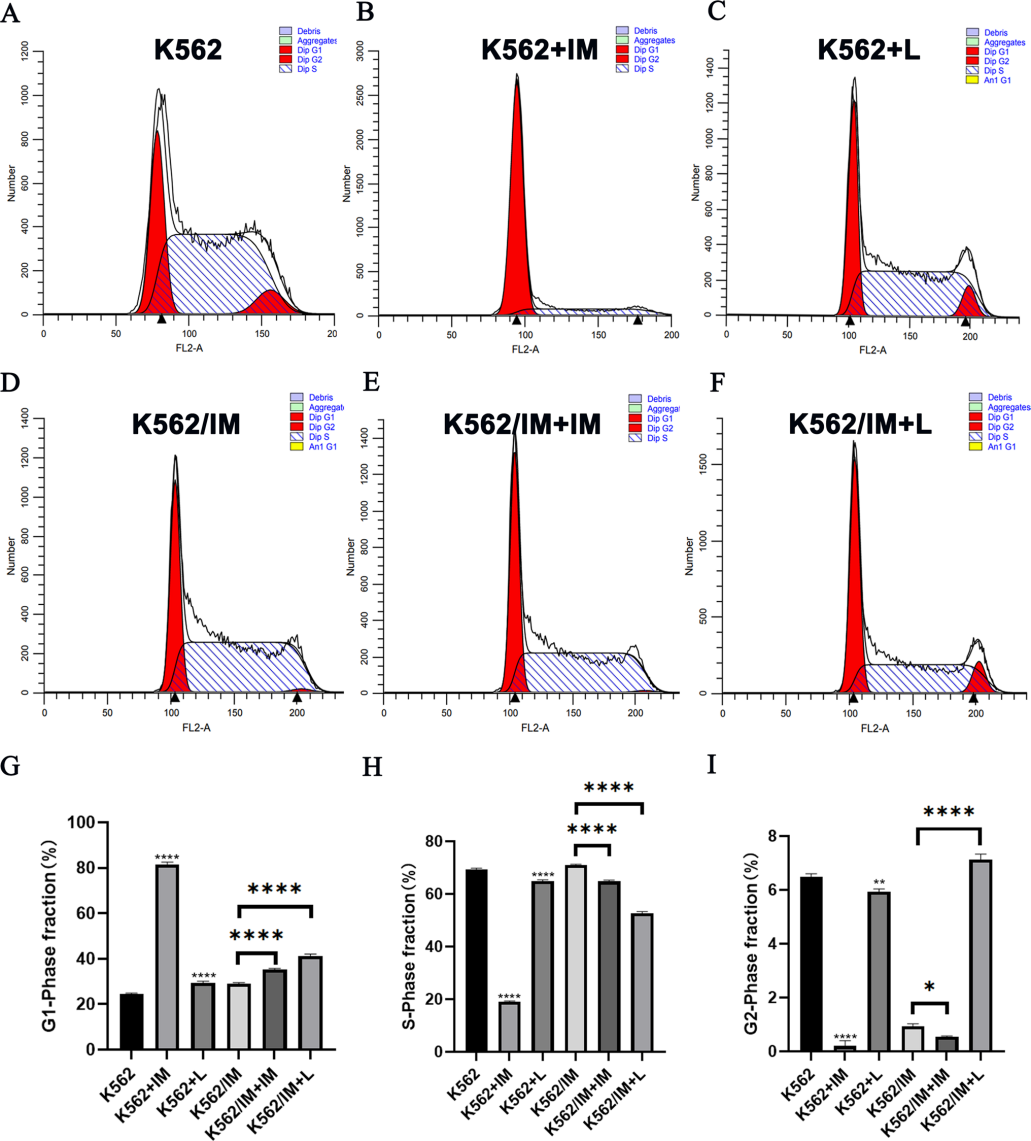
Lycorine induced K562 and K562/IM cell cycle arrest. **A–F** The cell cycle distribution of both K562 cells and K562/IM cells following a 24-h treatment with Lycorine (6 μM) and IM (5 μM) was assessed using flow cytometry, the groups include K562 group, K562+IM group, K562+Lycorine group, K562/IM group, K562/IM+IM group, K562/IM+Lycorine group. **G**-**I** Statistical analyses were conducted on the data pertaining to the cell cycle distribution among various dosage groups. *P < 0.05; **P < 0.01; ***P < 0.001; ****P < 0.0001

Flow cytometry was utilized to evaluate the cell cycle distribution of the aforementioned cells subsequent to a 24-h exposure to various drug regimens. Figure 4 illustrates the outcomes of treating K562 and K562/IM cells with 6 μM lycorine and 5 μM IM. In comparison to the control group, cells treated with either lycorine or IM in both K562 and K562/IM exhibited arrest in cell cycle progression at the G1 phase, accompanied by a modest reduction in the S phase. The K562 + L group demonstrated a modest reduction in the proportion of cells in the G2/M phase, whereas the K562/IM + L group displayed a significant elevation in the G2/M phase compared to the K562/IM group. The influence of IM on cell cycle arrest manifested more prominently in the K562 group than in the K562/IM group.

### Autophagy and apoptosis of K562 and K562/IM treated with lycorine

**Fig. 5:**
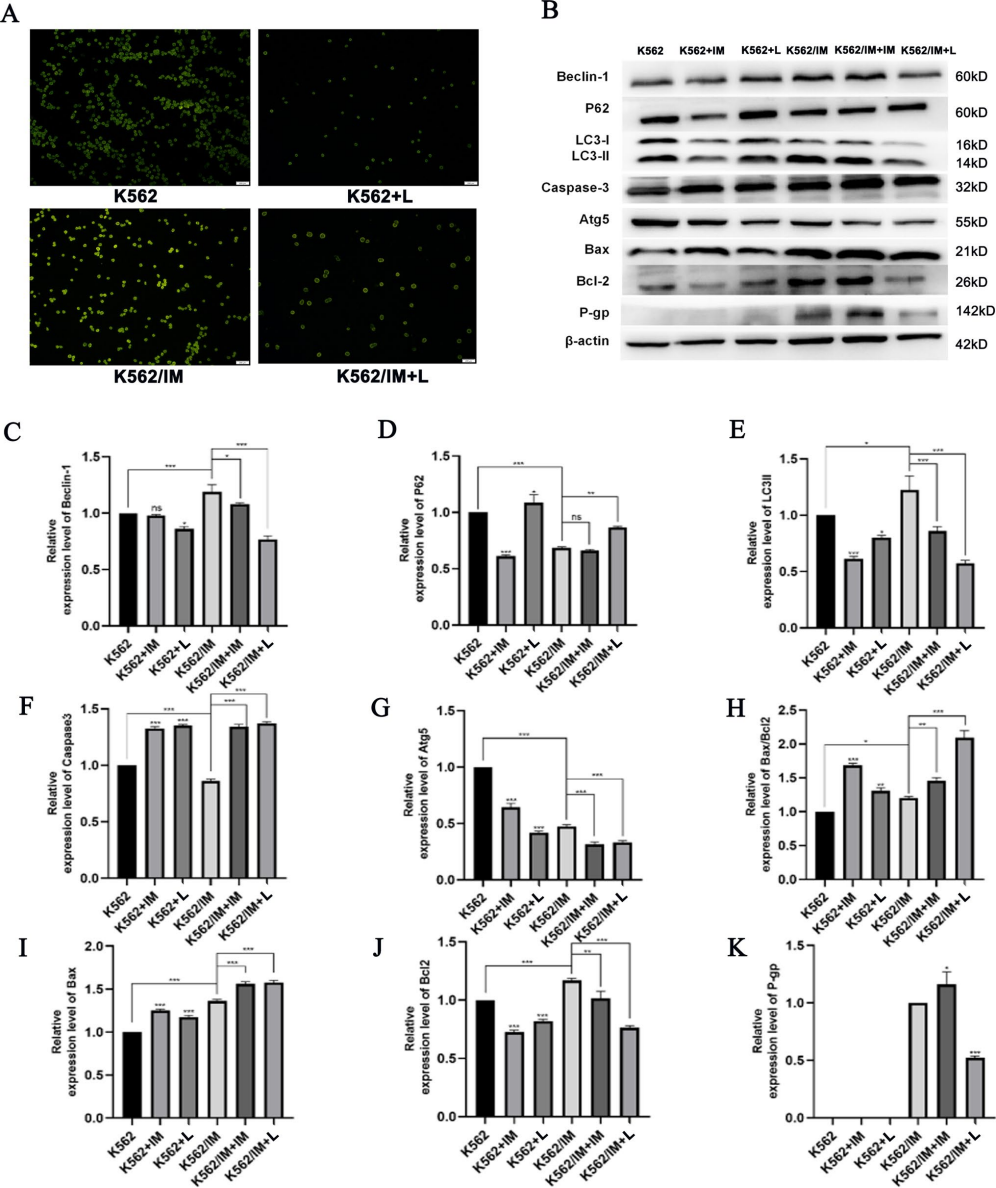
Autophagy and apoptosis of K562 and K562/IM treated with lycorine. **A** MDC staining experiment with different dosage groups for 72 h, including K562 group, K562+Lycorine group, K562/IM group and K562/IM+Lycorine group (Magnification 20×). **B** Different protein expression bands. **C** Gray value analysis diagram of Beclin-1. **D** Gray value analysis diagram of P62. **E** Gray value analysis diagram of LC3-II. **F** Gray value analysis of Caspase-3. **G** Gray value analysis diagram of Atg-5. **H** Gray value analysis of Bax/Bcl2. **I** Gray value analysis of Bax. **J** Gray value analysis of Bcl-2. **K** Gray value analysis of P-gp. *P < 0.05; **P < 0.01; ***P < 0.001; ****P < 0.0001

Figure 5A illustrates a substantial decrease in fluorescence intensity for both K562 and K562/IM cells following a 72-h exposure to 6 μM lycorine, providing clear evidence of lycorine's inhibitory impact on autophagy. Notably, the fluorescence intensity of K562/IM cells surpassed that of their normal K562 counterparts, indicative of an elevated autophagic level in the K562/IM resistant strains. This observation underscores the heightened autophagic activity associated with K562/IM resistance. Subsequently, we employed Western blot analysis to assess the expression levels of autophagy, apoptosis, and drug resistance-related proteins in K562 and K562/IM cells following a 24-h treatment with lycorine (6 μM) and IM (5 μM) (Fig. 5B). The alterations in protein expression are depicted in Fig. 5C–K. In comparison to the K562 group, the K562/IM group exhibited significant up-regulation in the expression of Beclin-1, LC3-II, and Bax/Bcl2, while the expression levels of Atg5, Caspase-3, and P62 were notably down-regulated. In the K562+IM group, P62 and LC3-II expressions were substantially reduced, whereas Caspase-3 and Bax/Bcl2 expressions were significantly increased. Within the K562+L group, P62, Caspase-3, and Bax/Bcl2 protein expressions were significantly up-regulated, and Atg-5 protein was down-regulated. Furthermore, compared with the K562 group, P-gp protein expression increased in the K562/IM group. In the K562/IM+IM group, Beclin-1, LC3-II, and Atg-5 protein expressions were down-regulated, while P62, Caspase-3, and Bax/Bcl2 protein expressions were significantly up-regulated compared to the K562/IM group. Similarly, the K562/IM+L group exhibited down-regulation in Beclin-1, LC3-II, Atg-5, and P-gp protein expressions, along with significant up-regulation in Caspase-3 and Bax/Bcl2 protein expressions. All observed differences were statistically significant.

### Lycorine killed K562 and K562/IM cells in vivo experiments

**Fig. 6:**
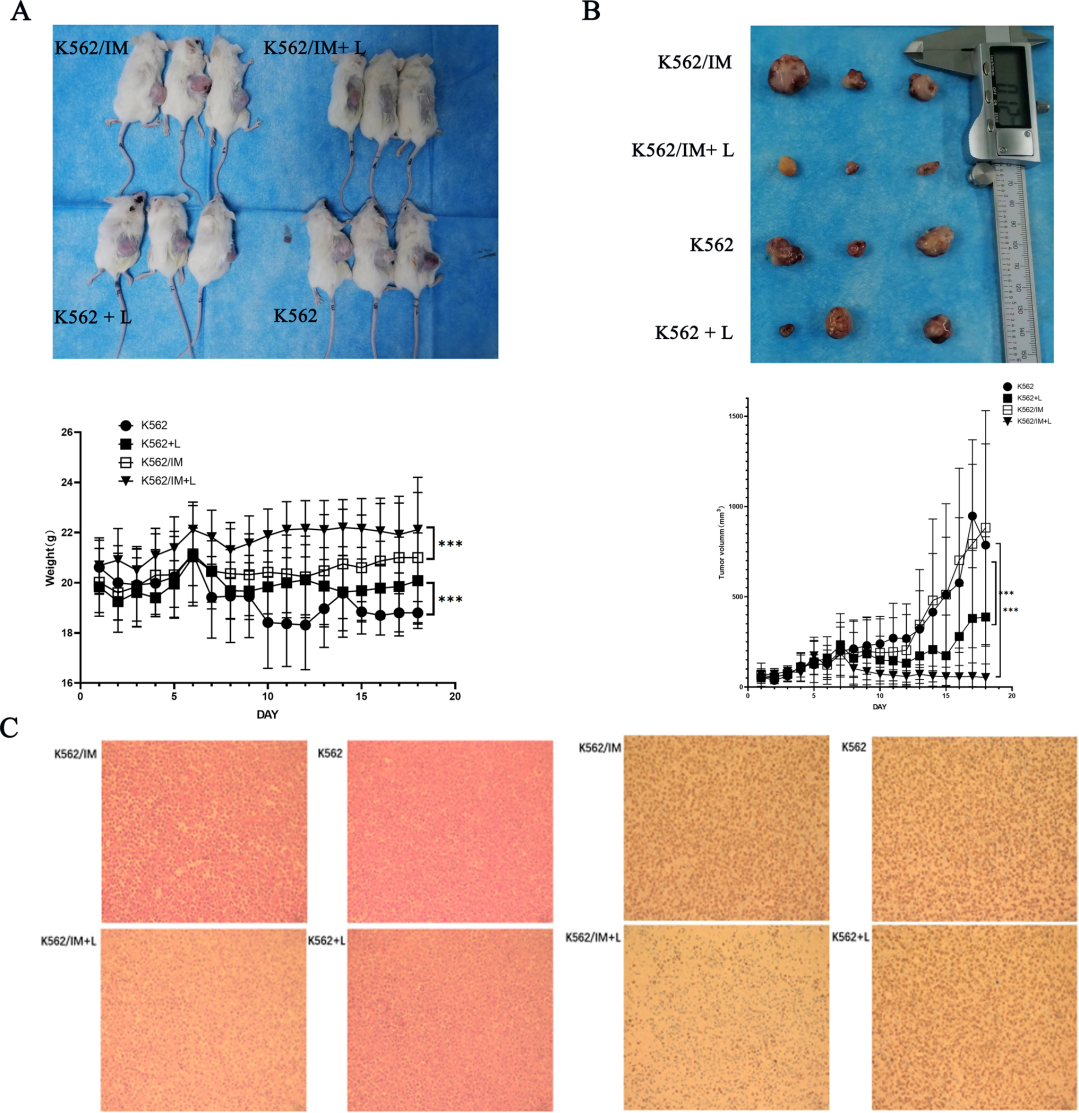
In vivo experiments in mice proved that lycorine killed K562 and K562/IM cells. **A **1 × 10^7 K562 or K562/IM cells were injected into NOD-SCID immunodeficient mice. The mice were randomized for intraperitoneal injection of lycorine at a dosage of 10 mg/kg/day. All tumors were removed, and the weight of tumors was measured for each group on the last day. **B** The appearance and volumm of mice tumor for each group. **C** Histological analysis using HE staining and Ki67 proliferation index staining indicated a reduced tumor cell area in the Lycorine treatment group compared to the K562 and K562/IM groups. *P < 0.05; **P < 0.01; ***P < 0.001; ****P < 0.0001

A murine model was established to encompass both K562 and a K562/IM drug-resistant variant through the subcutaneous inoculation of 1 × 10^7^ K562 or K562/IM cells into NOD-SCID immunodeficient mice over a 3–5-week period. The experimental cohort received intraperitoneal administration of lycorine at a dosage of 10 mg/kg/day, with continuous daily monitoring of mice's weight and tumor dimensions. After 17 days, euthanasia was performed, and tumor measurements were conducted. The outcomes, illustrated in Fig. 6A–B, revealed a significant reduction in tumor size for both K562 and K562/IM cells following 6 µM lycorine treatment (K562 + L, K562/IM + L), compared to the control group. Additionally, histological analysis using HE staining and Ki67 proliferation index staining indicated a diminished tumor cell area in the lycorine treatment group compared to the K562 and K562/IM groups (Fig. 6C). Notably, the drug-resistant K562/IM group exhibited a more pronounced increase in Ki67 post-treatment compared to the K562 group. However, no statistically significant change in Ki67 was observed following lycorine treatment in the K562 group.

## Discussion

CML is a hematopoietic stem cell-derived leukemia distinguished by the presence of the Philadelphia (Ph) chromosome translocation, leading to the formation of the BCR-ABL fusion gene. The BCR-ABL fusion protein is a tyrosine kinase with oncogenic properties. Presently, therapeutic approaches for individuals with CML encompass diverse TKIs. While TKIs have demonstrated efficacy in a substantial proportion of CML patients, there exists a need for further exploration and refinement of treatment modalities to address potential limitations and enhance overall patient outcomes [15], a significant proportion of individuals still harbor residual leukemia cells during prolonged medical treatment, with relapse anticipated upon discontinuation of TKI therapy [16]. Among these patients, about 20–30% may develop acquired resistance to long-term TKI administration [17]. The emergence of resistance, particularly in the case of TKIs like IM, significantly hampers their efficacy in the management of CML patients.

Autophagy plays a pivotal role in all stages of CML development. During the early stages of CML, autophagy exerts an anticancer effect, while in the middle to late stages, it assumes a pro-cancer and drug-resistant role. Particularly, during the blast crisis, autophagy protects CML cells, leading to increased proliferation and heightened mortality in patients transitioning to this critical phase. Consequently, the development of drugs targeting autophagy has emerged as a focal point in the research of therapeutic agents for chronic leukemias. Notably, research has demonstrated that lycorine effectively inhibits autophagy and exhibits robust anti-proliferative activity both in vivo and in vitro against multiple myeloma [18]. Furthermore, studies have unveiled lycorine's inhibitory effects on the proliferation of acute myeloid leukemia, B-cell acute lymphoblastic leukemia, and chronic lymphocytic leukemia cells [19–21]. Previous literature has reported that lycorine induces cell cycle arrest specifically in the G0/G1 phase of K562 cells [10]. Subsequent investigations in our study revealed a pronounced inhibitory impact of lycorine on the proliferation of both K562 and K562/IM cells. Beyond inducing cell cycle arrest, our findings demonstrated a significant up-regulation of Bax/Bcl-2, Bax, and Caspase-3, coupled with a notable down-regulation of Bcl-2. These molecular changes signify the effective induction of apoptosis in K562/IM cells by lycorine, rendering the cells susceptible to programmed cell death. This stands in stark contrast to imatinib's incapacity to induce apoptosis in K562/IM cells, underscoring the distinctive apoptotic response triggered by lycorine. Subsequently, we conducted mRNA-seq transcriptome sequencing analysis, revealing that differentially expressed genes are enriched in autophagy-related pathways such as PI3K/Akt, MAPK, and RAS, as evidenced by the results of KEGG signaling pathway enrichment analysis. In the advanced stages of CML, particularly in cases of IM resistance, autophagy becomes a driving force in furthering CML development. Consequently, our focus revolves around elucidating strategies to overcome TKI resistance by impeding autophagy and identifying novel therapeutic targets for CML.

The upregulation of Beclin-1 and LC3-II proteins in K562/IM, in comparison to normal K562 cells, concomitant with the downregulation of P62 protein, indicates an escalated autophagic activity in K562/IM. This heightened autophagic level in K562/IM was further confirmed by a significant increase in fluorescence intensity observed in MDC fluorescence staining compared to K562 cells. Consequently, it was deduced that the resistance of K562 to IM is intricately linked to an enhanced autophagic response. Following lycorine treatment, the expression levels of Beclin-1, Atg-5, LC3-II, Bcl-2, and P-gp in K562/IM markedly decreased, while the expression levels of Caspase-3, P62, Bax/Bcl-2, and Bax substantially increased. It is noteworthy that K562 inherently exhibits low P-gp expression, rendering it undetectable. Moreover, after lycorine treatment, the visible fluorescence intensity of both K562 and K562/IM cells decreased, indicating a partial inhibition of autophagy by lycorine. Additionally, it was observed that the combined treatment with the autophagy inducer APRA reduced the cytotoxic effect of lycorine on K562/IM, whereas the co-administration of the autophagy inhibitor HCQ increased the cytotoxicity of lycorine against K562 cells. Collectively, these findings unveil potential mechanisms underlying lycorine's ability to overcome K562/IM resistance through the inhibition of autophagy.

Beclin-1, an Atg6 homolog, plays a pivotal role as a positive regulator of autophagy in mammals [16]. The exclusive association of LC3-II, the only known protein linked to autophagosome quantity, with the membrane during autophagy signifies its direct correlation with the number of autophagosomes [22]. Throughout the autophagic process, membrane-bound LC3-II is specifically localized on the autophagosome membrane as it approaches closure. Acting as a regulatory component, p62 participates in the autophagosome assembly by bridging LC3 protein and ubiquitinated substrates. Subsequently, p62 undergoes degradation by autophagolysosomes during the middle and late stages [23]. The overall cellular expression level of p62 demonstrates an inverse correlation with autophagy activity [24]. In the conducted experiment, a reduction in the expression levels of Atg-5 and Beclin-1 proteins was observed. Overexpression of Bax resulted in the down-regulation of LC3-II and the up-regulation of P62. These alterations imply a decrease in autophagic bodies and a blockade of autophagic flow at this juncture, indicating that lycorine inhibits the initial phase of autophagy while concurrently suppressing autophagic levels in both K562 and K562/IM. Furthermore, in vivo experiments using NOD-SCID mice confirmed the effective inhibition of the growth of drug-resistant K562 cells by lycorine. The use of nanocarriers such as PLGA-PEG nanoparticles effectively enhances drug bioavailability and anti-tumor activity. Furthermore, there is a need for more quality improvement measures regarding treatment processes and outcomes [25, 26]. Nanocarrier technology, like PLGA-PEG nanoparticles, can be employed to improve drug bioavailability and targeting, which may hold significant importance for the treatment of CML patients. By loading current therapeutic drugs into nanoparticles, the stability and effectiveness of drugs in the body can be increased, reducing side effects and improving patients' quality of life. Immunomodulatory strategies may also be beneficial for the treatment of CML patients, particularly in addressing drug resistance and reducing the survival of leukemia stem cells [27, 28]. Additionally, CML patients often exhibit increased inflammatory responses, thus therapeutic approaches targeting inflammatory pathways may hold significant importance in improving patients' conditions and prognosis.

**Fig. 7:**
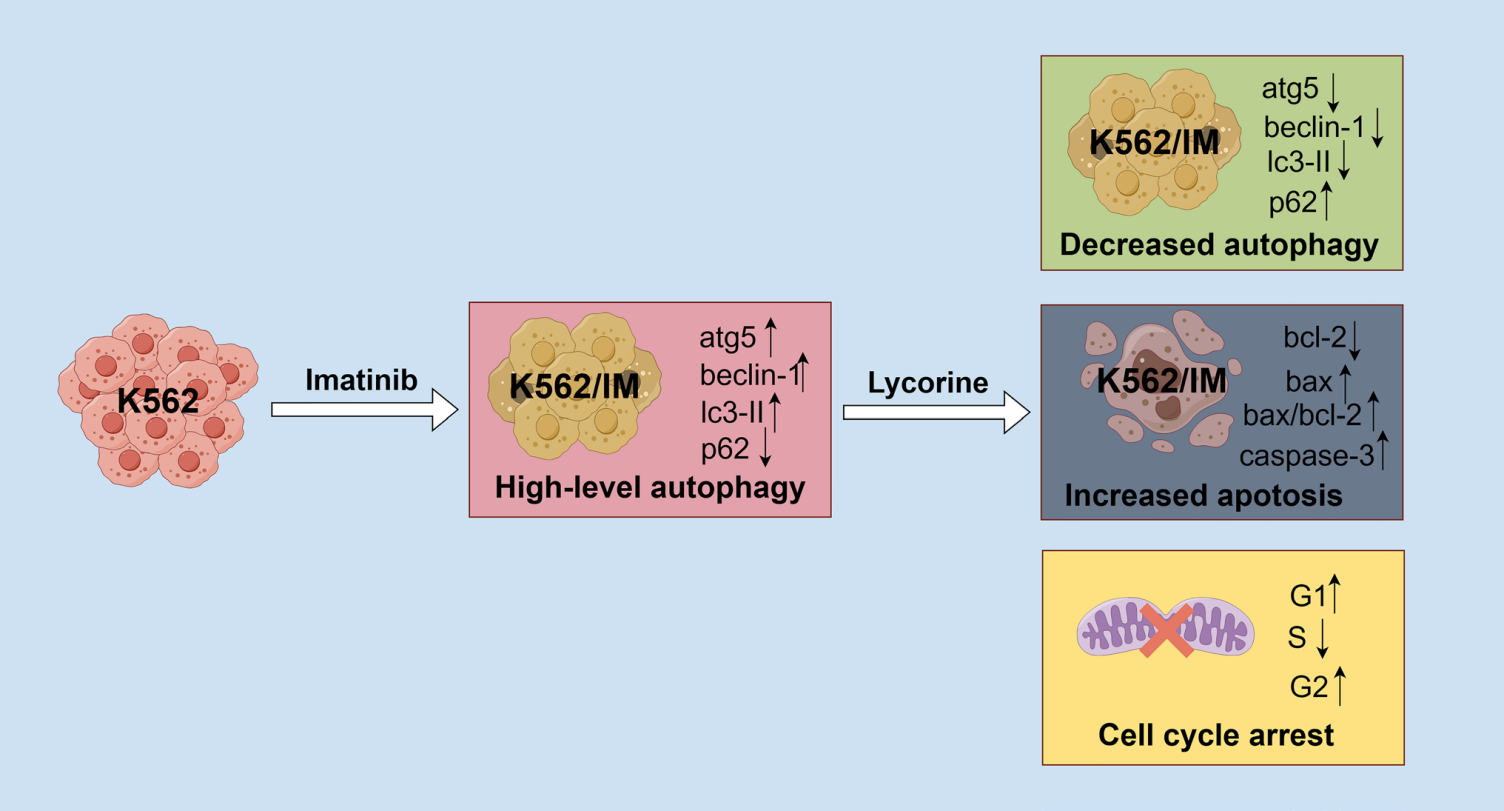
Lycorine attenuated proliferation by inducing apoptosis, inhibiting autophagy, and cell cycle arrest on K562/IM cell. Notably, K562/IM cells exhibited elevated levels of autophagy compared to K562 cells, and lycorine demonstrated efficacy in inhibiting the proliferation of K562/IM cells through multiple pathways, including the reduction of autophagic levels, promotion of apoptosis, and induction of cell cycle arrest

In conclusion, we successfully established a stable drug-resistant K562/IM cell line through a gradual increment in IM concentration. Subsequent bioinformatics analysis uncovered a potential association between IM resistance in K562 cells and autophagy. Notably, K562/IM exhibited elevated levels of autophagy compared to K562, and lycorine demonstrated efficacy in inhibiting the proliferation of K562/IM through multiple pathways, including the reduction of autophagic levels, promotion of apoptosis, and induction of cell cycle arrest (Fig. 7). In vivo experiments substantiated lycorine's ability to impede proliferation in both K562 and K562/M cells. This study represents the inaugural utilization of lycorine to investigate autophagy regulatory mechanisms in IM-resistant K562 cells, providing valuable insights into its influence on the autophagic cascade. The findings not only enhance the reliability of our results but also propose novel approaches to address TKI resistance. In summary, we offers a comprehensive exploration of lycorine as a potential treatment option for overcoming IM resistance in CML, highlighting its multifaceted mechanisms of action and providing valuable insights into novel therapeutic strategies for managing drug-resistant CML.

### Supplementary Information

Additional file1

## Data Availability

Data used in this study are available at corresponding author email. Sequence data that support the findings of this study have been deposited in the Gene Expression Omnibus with the accession GSE267522.
