# Supplementary figures and images for: Lycorine attenuated proliferation and induced apoptosis on imatinib-resistant K562 cell by inhibiting autophagy
Source: Discov Oncol. 2024 Jun 10;15:217. doi: 10.1007/s12672-024-01080-3 (PMC11164850; doi:10.1007/s12672-024-01080-3)

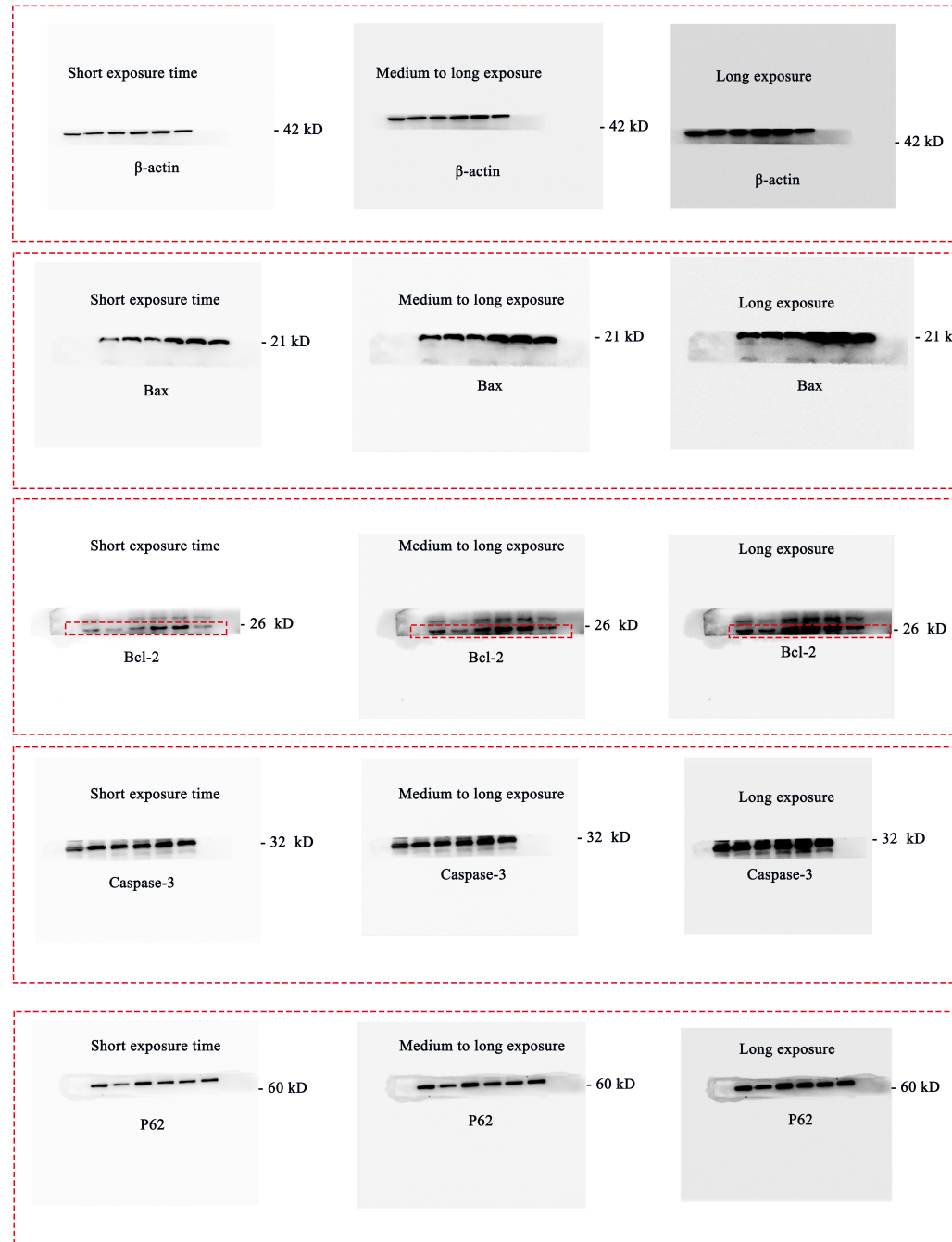

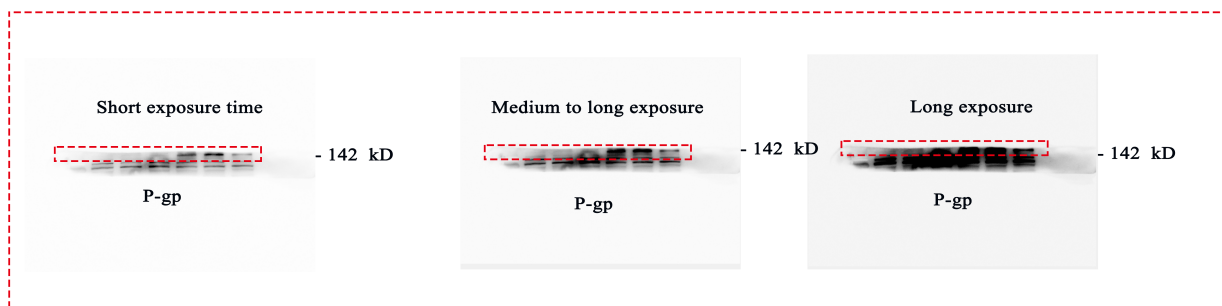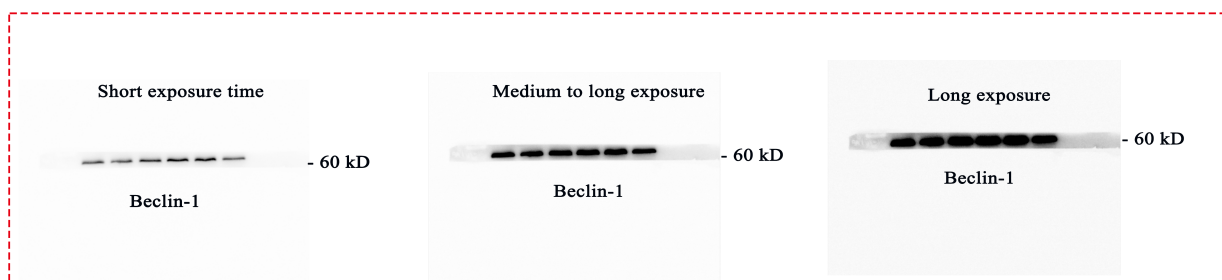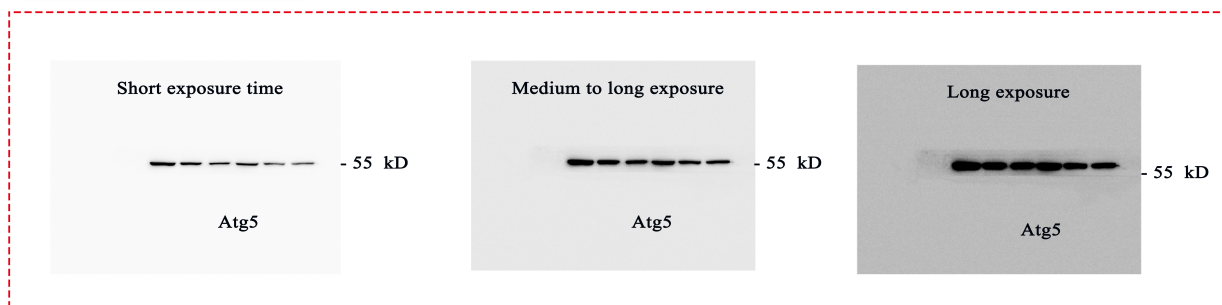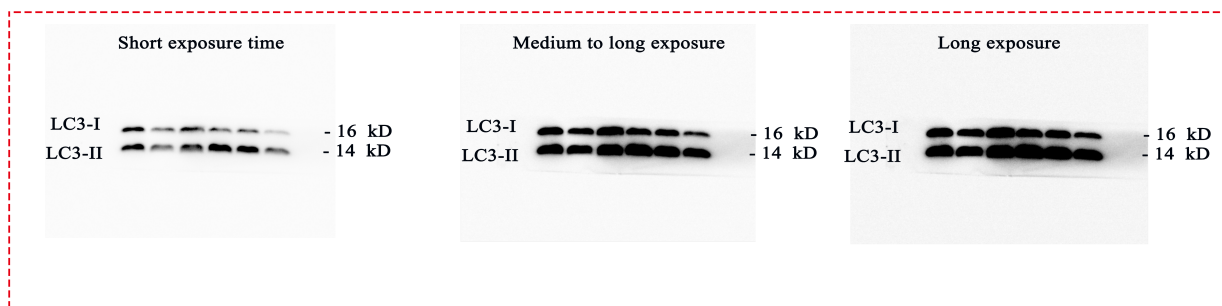

Supplement: Supplementary file 1 — Additional file1 [file 12672_2024_1080_MOESM1_ESM.pdf]
